# The ability to obtain, appraise and understand health information among undergraduate nursing students in a medical university in Chongqing, China

**DOI:** 10.1002/nop2.161

**Published:** 2018-05-21

**Authors:** Mao Zou, Yan Zhang, Fan Zhang, Ping Hu, Ruixue Bai, Wenjie Huang, Lu Lu, Abu S. Abdullah, Jeff Jianfei Guo, Manoj Sharma, Cesar Reis, Yong Zhao

**Affiliations:** ^1^ School of Public Health and Management Chongqing Medical University Chongqing China; ^2^ Research Center for Medicine and Social Development Chongqing Medical University Chongqing China; ^3^ Collaborative Innovation Center of Social Risks Governance in Health Chongqing Medical University Chongqing China; ^4^ Clinical Nutrition Department People’s Hospital of Deyang City Deyang China; ^5^ Medical Examination Center The Second Affiliated Hospital Chongqing Medical University Chongqing China; ^6^ Global Health Program Duke Kunshan University Kunshan China; ^7^ Duke Global Health Institute Duke University Durham North Carolina; ^8^ Department of General Internal Medicine Boston University Medical Center Boston Massachusetts; ^9^ Division of Pharmacy Practice and Administrative Sciences College of Pharmacy University of Cincinnati Medical Center Cincinnati Ohio; ^10^ Department of Behavioral and Environmental Health Jackson State University Jackson Mississipi; ^11^ Department of Preventive Medicine Loma Linda University Medical Center Loma Linda California

**Keywords:** appraise health information, Health Literacy Questionnaire, obtain health information, undergraduate nursing students, understand health information

## Abstract

**Aim:**

The aim of this study was to survey the ability of nursing students to obtain, appraise and understand health information and its influencing factors among undergraduate nursing students in a medical university in Chongqing, China.

**Design:**

A cross‐sectional survey.

**Method:**

The sample was obtained using stratified sampling methods. We used the internationally validated Health Literacy Questionnaire. Six hundred and fifteen (76.88%) of 800 nursing students completed participated anonymous questionnaires that measured their ability to obtain, appraise and understand health information.

**Results:**

Mean scores of nursing students to obtain, appraise and understand health information were 17.13, 13.07 and 17.78 respectively. Academic level, parental educational level and socioeconomic status were significantly associated with scores in obtaining, appraising and understanding health information.

## 1 INTRODUCTION

Health literacy is defined as the ability to obtain, process and use basic health information and services to make appropriate health decisions(Baker, 2006; Berkman, Sheridan, Donahue, Halpern, & Crotty, 2011). It is regarded by the World Health Organization (WHO) (2009) as a key factor for improving population health. The definition of health literacy not only implies the ability of a person to obtain and understand health information but also includes the ability to appraise and use health information to make appropriate health‐related decisions (Nutbeam, 2008; Sillence, Briggs, Harris, & Fishwick, 2007; Sørensen et al., 2012; Stonbraker, Befus, Nadal, Halpern, & Larson, 2016). Furthermore, the effect of health literacy on health outcomes begins with the ability to obtain information (Shieh, Mays, Mcdaniel, & Yu, 2009). Studies have shown that people with inadequate health literacy may have difficulty in obtaining and using information (Jeong & Kim, 2016; Spink & Cole, 2001). In turn, obtaining and understanding health information significantly influences a person’s ability to use health care, which results in better health outcomes and overall quality of life (Kelley, Su, & Britigan, 2016). Failure to properly obtain or understand health information negatively affects an individual’s health and can lead to health disparities (Custodio, Gard, & Graham, 2009; Eysenbach, Powell, Kuss, & Sa, 2002).

Patients with inadequate health literacy are unable to effectively communicate with health professionals, understand health information and choose appropriate treatment recommendations (Barrett & Puryear, 2006). When patients receive insufficient information to participate in decisions regarding their care, it limits the extent of their participation and thwarts early recovery. Healthcare professionals play a critical role in helping patients obtain and understand health information (Yeo, 2016). In addition, nursing professionals are often viewed as “communication brokers” between doctors and patients and are required to translate “medical language” into “everyday language” for patients (Bourhis, Roth, & Macqueen, 1989). Meanwhile, they are the main professionals responsible for patients’ health education and health promotion activities.

Moreover, with the increasing pressure exerted by an aging population and chronic non‐communicable diseases (Li et al., 2017), modern medical service model taking prevention as the centre gets more attention, asking residents to improve their health literacy. Healthcare institutions reform to create health promoting hospitals under the government’s plan for Health China 2030, which as a means of improving the health literacy of residents and addressing the burden of chronic non‐communicable diseases. It means that nurses’ service is not limited to patients in hospitals, but also to healthy people in communities and families. Therefore, nursing professionals need various types of health information to meet their clinical and educational needs (Dee & Stanley, 2005).

### 1.1 Background

Almost all nursing students will work as nurses; however, a previous study showed that 36.9% of nursing baccalaureate students were unaware that functional health literacy skills involve the ability to read, comprehend and make decisions on health care. Moreover, more than 60% of nursing baccalaureate students “never” or “sometimes” had experiences relating to health literacy (Cormier & Kotrlik, 2009). Another study showed that college students tend to lack access to health information (Syn & Kim, 2016). Our previous research showed that the medical students in Chongqing, southwestern China also had difficulty in obtaining, appraising and understanding health information (Zhang et al., 2016).

In China, some research work has been conducted to study the ability to obtain, appraise and understand health information among undergraduate nursing students. We found no research about this topic among undergraduate nursing students in a medical university in Chongqing, southwestern China. In this study, we aim to explore the ability to obtain, appraise and understand health information among undergraduate nursing students in medical university in Chongqing, southwestern China using the Health Literacy Questionnaire (HLQ), a survey tool with strong construct validity, reliability and acceptability (Osborne, Batterham, Elsworth, Hawkins, & Buchbinder, 2013).

## 2 THE STUDY

### 2.1 Aim

To survey the ability of nursing students to obtain, appraise and understand health information and its influencing factors by conducting a cross‐sectional study among undergraduate nursing students in a medical university in Chongqing, China.

### 2.2 Design

The study design and survey implementation in this study have been previously described (Zhang et al., 2016). A cross‐sectional study was conducted involving undergraduate nursing students of a medical university in Chongqing, China. Stratified cluster sampling was adopted, and the grade was used as the primary sampling unit. We chose nursing undergraduates in their 1, 2 and 3 Academic Years, each with four classes. Two nursing classes were randomly selected for Academic Year 1 and one nursing class was selected for Years 2 and 3.

### 2.3 Sample

In this study, these classes had a total of 800 nursing undergraduates, all of whom received a questionnaire; 721 questionnaires were received with a response rate of 90.125%. A total of 106 questionnaires were excluded from the analyses because of one of the following reasons: 1) in question 8 (What is your socioeconomic status?), the respondents answered that they did not know; 2) in question 9 (parent’s highest levels of education), the respondents answered that they did not know, or that this question was not suitable for them (e.g. some had no parents). 615 questionnaires were included in the analyses.

### 2.4 Ethical considerations

The Ethics Committee of Chongqing Medical University approved this study, with Preference Number 2015002. We obtained a written informed consent from all of the participants.

### 2.5 Questionnaire

HLQ was developed by Osborne et al. (Osborne et al., 2013) and the questionnaire has been shown to have strong construct validity, reliability and acceptability (Zhang et al., 2016). In this study, we obtained their permission and used the Chinese version of HLQ (Beauchamp et al., 2015). We re‐examined its reliability by assessing the internal reliability and the Cronbach’s alpha (α = 0.947). We used three domains (measured using one scale per domain) of the HLQ to explore the ability to obtain, appraise and understand health information among undergraduate nursing students. The three measures included: 1) Ability to find good health information; 2) Appraisal of health information; 3) Understanding health information enough to know what to do. Each measure contained five items. There were five response options for items pertaining ability to find good health information and understanding health information enough to know what to do and four response options for items pertaining to appraisal of health information.

To obtain the composite score, point sets were as follows for measures 1 and 3: cannot do = 1; very difficult = 2; quite difficult = 3; quite easy = 4; very easy = 5. Point sets for measure 2 were as follows: strongly disagree = 1; disagree = 2; agree = 3; strongly agree = 4. Hence, the full points possible for scales 1–3 were 25, 20 and 25 respectively.

### 2.6 Data analysis

These data were carefully checked before being entered into the database established using the Epi‐data 2.1 Software. SPSS 22.0 was used to analyse the data. Frequencies and percentages were calculated for count data, for example, demographic variables. Mean values and standard deviation were calculated for scores on obtaining, appraising and understanding health information. We set the low scores less than 60% of the total score and the high scores more than 80% of the total score. Chi‐square test was used to determine if frequencies had differences among different groups. T‐tests were used to compare the scores between two different groups. Analyses of variance (ANOVA) were performed to compare the scores among three different groups. However, for HLQ scales, while responses covered the full range of the scales, the assumptions of normal distribution were not met. And it also violated homogeneity of variances. We therefore used the robust analysis of variance (ANOVA) for analysis of HLQ scores using the Welch method. Where required, post hoc testing was undertaken using the Dunnett’s T3 method of multiple mean comparisons. We used multiple linear regression (MLR) model analysis to determine the factors associated with scores on obtaining, appraising and understanding health information. In the MLR, the scores were regarded as the dependent variable and age, sex, registered permanent residence, socioeconomic status, parent’s highest level of education, long‐term illness or disability and academic year were regarded as independent variables. The enter method was used to eliminate variables. Statistical significance was assumed as *p* < 0.05.

## 3 RESULTS

### 3.1 Demographic characteristics

Table 1 shows that among the participants (*N* = 615), most were female (90.6%), aged between 20 and 24 years (56.7%) and freshmen (51.3%); 52.5% were from rural areas.

**Table 1:** Demographic data for overall sample by academic year (*N* = 615)

		Academic year			
	Total	Year 1	Year 2	Year 3	
Variables	*N*(%)	*N*(%)	*N*(%)	*N*(%)	*p*
Age					0.0001
15–19 years old	266 (43.3%)	224 (70.9%)	40 (23.5%)	2 (1.6%)	
20–24 years old	349 (56.7%)	92 (29.1%)	130(76.5%)	127 (98.4%)	
Sex					0.103
Male	58 (9.4%)	37 (11.7%)	10 (5.9%)	11 (8.5%)	
Female	557 (90.6%)	279 (88.3%)	160 (94.1%)	118 (91.5%)	
Registered permanent residence					0.003
Urban area	292 (47.5%)	129 (40.8%)	94 (55.3%)	69 (53.5%)	
Rural area	323 (52.5%)	187 (59.2%)	76 (44.7%)	60 (46.5%)	
Socioeconomic status					0.063
Below average	282 (45.9%)	155 (49.1%)	65 (38.2%)	62 (48.1%)	
Average or higher	333 (54.1%)	161 (50.9%)	105 (61.8%)	67 (51.9%)	
Parent’s highest levels of education					0.051
Has not completed high school/secondary school	390 (63.4%)	204 (64.6%)	96 (56.5%)	90 (69.8%)	
Completed high school/secondary school	225 (36.6%)	112 (35.4%)	74 (43.5%)	39 (30.2%)	
Long‐term illness or disability					0.0001
Yes	55 (8.9%)	16 (5.1%)	27 (15.9%)	12 (9.3%)	
No	560 (91.1%)	300 (94.9%)	143 (84.1%)	117 (90.7%)	

We classified the participants into three groups according to their academic year, namely, Years 1, 2 and 3. Table 1 shows that the proportion of females was more than 88.0% in every academic year. More than half of the participants in each academic year believed that their socioeconomic status was average or higher and less than half of the participants in every academic year responded that their parents had completed high school or secondary school. We found that age (*p* < 0.0001), registered permanent residence (*p* = 0.003) and long‐term illness or disability (*p* < 0.0001) significantly varied across different academic years.

### 3.2 Scores for obtaining, appraising and understanding health information

In “ability to find good health information,” and “understanding health information well enough to know what to do,” 25 points were full points. In “appraisal of health information,” 20 points was full point. Mean scores for each scale and items under each measure are shown in Table 2. Overall, the mean scores of measures 1–3 were as follows: 17.13 (*SD* 3.25), 13.07 (*SD* 2.31) and 17.78 (*SD* 3.03). In each of the measure and items pertaining to that measure, the scores in year 3 were highest, whereas the scores in year 1 were lowest. We also found that all mean scores in year 1 were lower than the average score in total participants. The percentage of having different levels of difficulty or selecting “disagree” were lowest in year 3 and highest in year 1. The differences were statistically significant (*p* < 0.0001).

**Table 2:** Average scores of each scale and item, and percentage of feeling different levels of difficulty or level of disagreement in different academic year

				Academic year								
	Total			Year 1			Year 2			Year 3		
Scale	M	SD	N (%)	M	SD	N (%)	M	SD	N (%)	M	SD	N (%)
Scale 1. Ability to find good health informationRange 1–5 (1 = cannot do 2 = very difficult 3 = quite difficult 4 = quite easy 5 = very easy)	17.13	3.25		15.37	3.34		18.28	1.72		19.95	1.37	
Find information about health problems	3.58	0.83	222 (36.1%)	3.25	0.91	171 (54.1%)	3.81	0.60	41 (24.1%)	4.06	0.46	10 (7.8%)
Find health information from several different places	3.46	0.85	265 (43.1%)	3.13	0.92	194 (61.4%)	3.69	0.64	53 (31.2%)	3.96	0.49	18 (14.0%)
Get information about health so you are up to date with the best information	3.21	0.85	360 (58.5%)	2.87	0.87	242 (76.6%)	3.36	0.67	96 (56.5%)	3.88	0.52	22 (17.1%)
Get health information in words you understand	3.42	0.90	270 (43.9%)	3.00	0.94	209 (66.1%)	3.71	0.61	53 (31.2%)	4.06	0.50	8 (6.2%)
Get health information by yourself	3.46	0.82	260 (42.3%)	3.11	0.92	200 (63.3%)	3.71	e0.49	50 (29.4%)	3.99	0.42	10 (7.8%)
Scale 2. Appraisal of health informationRange 1–4 (1 = strongly disagree 2 = disagree 3 = agree 4 = strongly agree)	13.07	2.30		12.39	2.37		13.11	1.60		14.67	2.16	
I compare health information from different sources	2.72	0.68	210 (34.1%)	2.57	0.69	135 (42.7%)	2.79	0.58	50 (29.4%)	2.98	0.68	25 (19.4%)
When I see new information about health, I check up on whether it is true or no	2.66	0.67	242 (39.3%)	2.56	0.70	146 (46.2%)	2.65	0.56	63 (37.1%)	2.91	0.69	33 (25.6%)
I always compare health information from different sources and decide what is best for me	2.60	0.67	259 (42.1%)	2.49	0.71	160 (50.6%)	2.57	0.56	75 (44.1%)	2.94	0.58	24 (18.6%)
I know how to find out if the health information I receive is right or not	2.56	0.65	274 (44.6%)	2.43	0.65	172 (54.4%)	2.53	0.55	78 (45.9%)	2.93	0.63	24 (18.6%)
I ask healthcare providers about the quality of the health information I find	2.53	0.69	293 (47.6%)	2.35	0.69	190 (60.1%)	2.58	0.55	73 (42.9%)	2.91	0.66	30 (23.3%)
Scale 3. Understanding health information well enough to know what to doRange 1–5 (1 = cannot do 2 = very difficult 3 = quite difficult 4 = quite easy 5 = very easy)	17.78	3.03		16.00	2.97		18.99	1.33		20.56	1.53	
Confidently fill medical forms in the correct way	3.51	0.88	269 (43.7%)	3.21	0.96	190 (60.1%)	3.69	0.65	60 (35.3%)	4.02	0.64	19 (14.7%)
Accurately follow the instructions from healthcare providers	3.67	0.84	203 (33.0%)	3.38	0.92	156 (49.4%)	3.89	0.60	34 (20.0%)	4.12	0.58	13 (10.1%)
Read and understand written health information	3.66	0.79	188 (30.6%)	3.36	0.89	153 (48.4%)	3.88	0.51	32 (18.8%)	4.12	0.47	3 (2.3%)
Read and understand all the information on medication labels	3.42	0.92	295 (48.0%)	3.08	0.93	202 (63.9%)	3.53	0.67	81 (47.6%)	4.09	0.78	12 (9.3%)
Understand what healthcare providers are asking you to do	3.52	0.81	243 (39.5%)	2.97	0.77	243 (76.9%)	4.00	0.00	0 (0%)	4.21	0.41	0 (0%)

M, mean; *SD*, standard deviation.

### 3.3 Ability to find good health information

Overall, Table 2 shows that in terms of “ability to find good health information”, the lowest mean score was in “getting information about health so you are up to date with the best information” (mean score: 3.21, *SD* 0.85) and 58.5% participants answered that they had different levels of difficulty in obtaining health information. The highest mean score was in “finding information about health problems” (mean score: 3.58, *SD* 0.83), whereas 36.1% participants thought that they had different levels of difficulty in “finding information about health problems.” In different grades, the lowest and highest mean score were the same as the overall score except for the highest mean score in year 3, which was in “finding information about health problems” and “getting health information in words you understand.”

### 3.4 Appraisal of health information

Mean scores for five items pertaining to “appraisal of health information” were shown in Table 2. Overall, the highest mean score was in “I compare health information from different sources” (mean score: 2.72, *SD* 0.68). However, 34.1% of the participants indicated that they could not compare health information from different sources. The lowest mean score was in “I ask healthcare providers about the quality of the health information I find” (mean score: 2.53, *SD* 0.69) and 47.6% of the participants answered that they had difficulty in asking health care providers about the quality of the health information they find. However, 54.4% of year 2 students had different levels of difficulty in how to find out if the health information they received was right or not.

### 3.5 Understanding health information well enough to know what to do

The lowest mean scores for five items pertaining to “understanding health information well enough to know what to do” was in “reading and understanding all the information on medication labels” (mean score: 3.42, *SD* 0.92) and 48.0% participants answered that they had difficulty in it. However, the lowest mean scores in year 1 was “understanding what healthcare providers are asking you to do,” with 76.9% of year 1 students reporting difficulty in it, whereas the scores in year 2 and 3 were highest, with everyone reporting ease in understanding what healthcare providers were asking them to do.

Furthermore, Table 3 shows that compared with participants who were 15–19 years old, the participants aged 20–24 year old were more likely to have higher scores in obtaining, appraising and understanding health information. All the differences were statistically significant (*p* < 0.0001). In addition, the higher the academic year, the higher the scores in obtaining, appraising and understanding health information. All the differences were statistically significant (*p* < 0.0001). Participants with average or higher socioeconomic status were more likely to have higher scores in “ability to find good health information” and “understanding health information well enough to know what to do.” The differences were statistically significant (*p* < 0.0001 and *p* < 0.0001 respectively). Those participants with parents that completed high school scored higher than nursing students with parents that had not completed high school in obtaining, appraising and understanding health information. The differences were statistically significant (*p* = 0.001, *p* = 0.030 and *p* < 0.0001 respectively).

**Table 3:** Association between scores and demographic characteristics (M±*SD*)

	Ability to find good health information		Appraisal of health information		Understanding health information well enough to know what to do	
	M	*SD*	M	*SD*	M	*SD*
Age						
15–19 years old	15.80	3.43	12.45	2.37	16.54	3.01
20–24 years old	18.15	2.71	13.54	2.15	18.73	2.69
*t*	−9.212^a^		−5.916		−9.365^a^	
*p*‐value	0.0001		0.0001		0.0001	
Sex						
Male	16.66	4.00	12.78	2.18	17.38	3.63
Female	17.18	3.17	13.10	2.32	17.82	2.96
*t*	−0.9878^a^		−1.015		−0.898	
*p*‐value	0.332		0.311		0.372	
Registered permanent residence						
Urban area	17.27	3.40	13.07	2.49	18.01	3.21
Rural area	17.02	3.11	13.07	2.13	17.57	2.85
t	0.958		0.037^a^		1.819	
*p*‐value	0.338		0.971		0.069	
Socioeconomic status						
Below average	16.52	3.57	12.87	2.38	17.11	3.51
Average or higher	17.65	2.86	13.23	2.42	18.35	2.42
*t*	−4.264^a^		−1.944		−4.979^a^	
*p*‐value	0.0001		0.052		0.0001	
Parent’s highest levels of education						
No high school	16.81	3.42	12.92	2.36	17.24	3.18
Completed high school	17.69	2.86	13.33	2.19	18.72	2.48
*t*	−3.418^a^		−2.171		−6.450^a^	
*p*‐value	0.001		0.030		0.0001	
Long‐term illness or disability						
Yes	18.11	3.63	13.00	1.98	18.80	3.90
No	17.04	3.20	13.08	2.34	17.68	2.92
*t*	2.336		−0.230		2.623	
*p*‐value	0.02		0.818		0.009	
Academic year						
Year 1	15.37	3.34	12.39	2.37	16.00	2.97
Year 2	18.28	1.72	13.11	1.60	18.99	1.33
Year 3	19.95	1.37	14.67	2.16	20.558	1.53
*F*	212.547^a^		47.958^a^		226.246^a^	
*p*‐value	0.0001		0.0001		0.0001	

M, mean; *SD*, standard deviation.

a Values with results were tested using Satterthwaite *t* test and robust ANOVA respectively.

### 3.6 Factors Associated with scores in obtaining, appraising and understanding health information

The results of the MLR analysis are shown in Table 4. We found that registered permanent residence, socioeconomic status, parent’s highest level of education and academic year were positively correlated with the scores in obtaining, appraising and understanding health information.

**Table 4:** Factors Associated with Scores in obtaining, appraising, and understanding health information

	Ability to find good health information			Appraisal of health information			Understanding health information well enough to know what to do		
Variable	(R_adj_^2^ = 0.387, *p *<* *0.0001)			(R_adj_^2^ = 0.158, *p *<* *0.0001)			(R_adj_^2^ = 0.487, *p *<* *0.0001)		
β	*SE*	*p*‐value	β	*SE*	*p*‐value	β	*SE*	*p*‐value
Age									
15–19 years old vs. 20–24 years old	0.161	0.261	0.539	0.193	0.217	0.374	−0.046	0.223	0.836
Sex									
Male vs. female	0.288	0.359	0.422	0.249	0.298	0.404	0.215	0.306	0.482
Registered permanent residence									
Urban area vs. rural area	0.796	0.225	**0.0001**	0.405	0.187	**0.031**	0.766	0.192	**0.0001**
Socioeconomic status									
Below average vs. average or higher	1.111	0.218	**0.0001**	0.394	0.181	**0.030**	1.117	0.186	**0.0001**
Parent’s highest levels of education									
No high school vs. completed high school	0.970	0.222	**0.0001**	0.566	0.184	**0.002**	1.553	0.189	**0.0001**
Long‐term illness or disability									
With vs. without	0.525	0.371	0.157	−0.193	0.308	0.531	0.510	0.316	0.107
Academic year									
Year 1 (Reference)									
Year 2	2.682	0.281	**0.0001**	0.605	0.234	**0.010**	2.810	0.240	**0.0001**
Year 3	4.584	0.326	**0.0001**	2.218	0.271	**0.0001**	4.732	0.278	**0.0001**

*p* < 0.05 means statistically significant.

## 4 DISCUSSION

Health literacy is regarded as a key factor for improving population health (WHO, 2009) and it indicates the ability to obtain, appraise and understand health information (Nutbeam, 2008; Sillence et al., 2007; Sørensen et al., 2012; Stonbraker et al., 2016). As a future service provider for medical and health systems, nursing students will act as a “bridge” between patients and doctors in patients’ health education and health promotion activities. A nursing students’ ability to obtain, appraise and understand health information not only affects their future health, but also affects the quality of service they provide in.

In this study, we explored the ability to obtain, appraise and understand health information among undergraduate nursing students in a medical university of Chongqing, China using HLQ. Compared with a survey in Australia, which also used HLQ (Beauchamp et al., 2015), the mean scores in our study were not high. The mean score on “ability to find good health information” was 17.13 (total score: 25), indicating that the participants had difficulty in accessing health information when required and they depended on others to offer information. In “appraisal of health information,” the mean score was 13.07 (total score: 20), indicating that no matter how hard they try, they cannot understand most health information and get confused when information is conflicting. In “understanding health information well enough to know what to do,” the mean score was 17.78 (total score: 25), which showed that they had problems understanding written health information or instructions related to treatments or medications. Furthermore, they had problems with reading or writing when completing medical forms.

Previous studies have shown that the ability to obtain, appraise and understand health information were closely related to education and socioeconomic status (Bjarnadottir, Millery, Fleck, & Bakken, 2016; Jeong & Kim, 2016; Koo, Lu, & Lin, 2016; Norman & Skinner, 2006; Özkan, Mellema, Nazzal, Lee, & Ring, 2016). Consistent with previous studies, we also found that academic year and socioeconomic status were positively correlated with the ability to obtain, appraise and understand health information. With the increase in academic year, the scores in obtaining, appraising and understanding health information were higher. Senior students learned more medical knowledge and knew how to find health information. They could appraise and understand health information, thereby proving the importance of school education. Moreover, participants who believed that their socioeconomic status was average or higher were more likely to have higher scores than those who believed their socioeconomic status was below average. This may be because there is more exposure and access to educational information from parents or other health care sources for students with a higher socioeconomic status. In our study, participants were students whose socioeconomic status reflected their parents’ socioeconomic status. In other words, parents’ socioeconomic status was one of the influencing factors. In addition, we found that parents’ educational attainment was positively correlated with the students’ ability to obtain, appraise and understand health information. This may be because parents with a higher degree of education are more aware of health knowledge, can master advanced health concepts and can better guide their children in the learning process. This finding suggests that parental education plays an important role in improving the ability of students to obtain, appraise and understand health information. Consistent with previous studies (Dutta‐Bergman, 2005; Houston & Allison, 2002; Wald, Dube, & Anthony, 2007), we found that participants with long‐term illness or disability can easily understand health information. They pay more attention to their health and are more likely to contact healthcare providers to learn more about their health concerns. Thus, they can easily understand health information.

This study found that the ability to obtain, appraise and understand health information among undergraduate nursing students was suboptimal. In addition, we found that school education played a key role. Previous research also suggested that education on how to access health information should also be provided (Jeong & Kim, 2016). Universities in China should actively improve the educational curriculum of the undergraduate nursing student. Individuals that train undergraduate nurses should pay more attention to improving the ability to obtain, appraise and understand health information. Such improvements could be achieved by developing the training program and incorporating relevant educational components into the undergraduate nursing education curriculum. Meanwhile, it is necessary to strengthen the management and cultivate an environment aimed at improving a nursing students’ ability to use health information. The incorporation of such educational components should be evaluated before they are applied at a national scale.

### 4.1 Limitations

Nonetheless, this study had some limitations. First, large differences existed in the numbers of females and males. However, nursing studies show that this observation is a reality in China, where male students do not prefer to become nurses. Given the large gender gap in the study population, the results cannot represent the ability of male nursing students to obtain, appraise and understand health information. Second, we only surveyed freshmen, sophomore and junior nursing students and did not investigate higher educational levels (graduate, master, or doctoral students), or grades beyond the junior level. Third, the data obtained through the cross‐sectional survey does not provide direct causal inferences to explore whether unmeasured factors may better explain the observed relationships and it cannot determine the direction of the causality.

## 5 CONCLUSION

Overall, we found that the ability to obtain, appraise and understand health information among undergraduate nursing students was suboptimal. Education played a key role. Medical universities, therefore, should incorporate an educational component into the curriculum that will cultivate nursing students’ ability to use health information.

## CONFLICTS OF INTEREST

The authors have declared that no competing interests exist.

## AUTHOR CONTRIBUTIONS

YZ, FZ, YZ: Conceived and designed the experiments. YZ, PH, RB, WH, LL: Performed the experiments. MZ, YZ: Analysed the data. YZ, MZ: wrote the paper. FZ, YZ, ASA, JJG, MS, CR: Critically reviewed the paper.
